# Effects of the Multidisciplinary Risk Assessment and Management Program for Patients with Diabetes Mellitus (RAMP-DM) on biomedical outcomes, observed cardiovascular events and cardiovascular risks in primary care: a longitudinal comparative study

**DOI:** 10.1186/s12933-014-0127-6

**Published:** 2014-08-21

**Authors:** Fang Fang Jiao, Colman Siu Cheung Fung, Carlos King Ho Wong, Yuk Fai Wan, Daisy Dai, Ruby Kwok, Cindy Lo Kuen Lam

**Affiliations:** Department of Family Medicine and Primary Care, Li Ka Shing Faculty of Medicine, The University of Hong Kong, 3/F Ap Lei Chau Clinic, 161 Main Street, Ap Lei Chau, Hong Kong; Primary and Community Services, Hospital Authority Head Office, Hong Kong Hospital Authority, Hospital Authority Building, 147B Argyle Street, Kowloon, Hong Kong

**Keywords:** RAMP-DM, Multidisciplinary intervention, Risk stratification, Effectiveness, Diabetes

## Abstract

**Background:**

To assess whether the Multidisciplinary Risk Assessment and Management Program for Patients with Diabetes Mellitus (RAMP-DM) led to improvements in biomedical outcomes, observed cardiovascular events and predicted cardiovascular risks after 12-month intervention in the primary care setting.

**Methods:**

A random sample of 1,248 people with diabetes enrolled to RAMP-DM for at least 12 months was selected and 1,248 people with diabetes under the usual primary care were matched by age, sex, and HbA_1_c level at baseline as the usual care group. Biomedical and cardiovascular outcomes were measured at baseline and at 12-month after the enrollment. Difference-in-differences approach was employed to measure the effect of RAMP-DM on the changes in biomedical outcomes, proportion of subjects reaching treatment targets, observed and predicted cardiovascular risks.

**Results:**

Compared to the usual care group, RAMP-DM group had lower cardiovascular events incidence (1.21% vs 2.89%, *P* = 0.003), and net decrease in HbA_1_c (−0.20%, *P* < 0.01), SBP (−3.62 mmHg, *P* < 0.01) and 10-year cardiovascular disease (CVD) risks (total CVD risk, −2.06%, *P* < 0.01; coronary heart disease (CHD) risk, −1.43%, *P* < 0.01; stroke risk, −0.71%, *P* < 0.01). The RAMP-DM subjects witnessed significant rises in the proportion of reaching treatment targets of HbA1c, and SBP/DBP. After adjusting for confounding variables, the significance remained for HbA_1_c, predicted CHD and stroke risks.

**Conclusions:**

The RAMP-DM resulted in greater improvements in HbA_1_c and reduction in observed and predicted cardiovascular risks at 12 months follow-up, which indicated a risk-stratification multidisciplinary intervention was an effective strategy for managing Chinese people with diabetes in the primary care setting.

**Trial registry:**

ClinicalTrials.gov, NCT02034695

## Background

Diabetes mellitus (DM) has become one of the major disease burdens in the world. It was estimated that by 2013, there were at least 382 million people with diabetes all over the world [1], and a 55% increase is anticipated by 2035 [2]. In Hong Kong, the prevalence of diagnosed diabetes reached 5% in the whole population and 20% in those aged above 65 years old. Diabetes is the leading cause of coronary heart disease, stroke, end stage renal disease, blindness and amputation [3,4]. Early screening of the risk factors or precursory symptoms of diabetic complications is crucial, so early treatment can be given to prevent complications and mortalities. Many international guidelines have started to emphasis the comprehensive care for people with diabetes, which takes into account the integrated management of microvascular and macrovascular risk rather than only focusing on glycemic control [5–7].

Given the large number of people with diabetes and the huge disease burden, risk stratification based management strategy is appealing, so that resources can be allocated accordingly. Personalized treatment goals in clinical measurements and lifestyle modifications based on risk stratification are highly recommended by current guidelines [6–8]. Personalized diabetology is advocated by researchers as the potential solution to deal with the uncertainty in treatment and translate the evidence from randomized control trials (RCTs) to the real-world. However, studies on the effects of personalized treatment are sparse [9].

The Chronic Care Model in primary care developed by Dr. Wagner provided a multidimensional solution to achieving optimal primary care for patients with chronic diseases [10]. Among the six components identified in this model, four are usually explicitly addressed in chronic care management, which are delivery system redesign, decision support, clinical information system and self-management support. A systematic review found that to achieve positive outcomes, at least two of these components were included in interventions, and positive outcomes were found in all five studies containing four components [11], indicating that multidisciplinary approach is crucial to improve the control of diabetes.

Previously, a prospective trial of risk stratification and intervention involving 370 people with diabetes was completed in the U.S. [12]. Risk stratification was based on blood pressure, self-monitoring of blood glucose, microalbuminuria, foot examination and self-reported complications. Interventions for high-, moderate- and low-risk groups followed the American Diabetes Association Clinical Practice Recommendations. After a 12-month follow-up, this study found a considerable increase in the percentage of patients reaching HbA_1_C < 7% and blood pressure <130/85 mmHg. The net benefit of the intervention group compared to the control group was not reported [12]. Although the intervention in this study involves all the four components of the Chronic Care Model [10], it was conducted within staff-model primary care clinics. The generalizability of the study results to Asian populations is, however, doubtful.

In Asia, researchers of the Joint Asia Diabetes Evaluation Program (JADE) developed a web-based risk stratification and comprehensive care model for people with diabetes. This model includes a series of risk engines to stratify patients into different risk groups, and comprehensive care protocols recommended by the International Diabetes Federation. Doctors can access to this care model by an electronic portal [13]. This program addressed the decision support and clinical information system. It was reported that 3687 patients over seven Asian countries were enrolled through the electronic portal during 2007 to 2009, whereas the effectiveness of this model compared to the usual care is not clear [14].

A complex integrated care program for people with diabetes and the elderly was piloted in London. The interventions included risk stratification and a multi-disciplinary team led by general practitioners or consultants [15]. A comprehensive evaluation approach was proposed to assess the impact of the program on clinical outcomes as well as service use [16]. The results of the evaluation have not reported yet.

Up to date, evidence is lacking about the effectiveness of risk-stratification and risk-specific management programs for Chinese people with diabetes in the primary care setting. This study aimed at evaluating the effectiveness of a Multidisciplinary Risk Assessment and Management Program for Patients with Diabetes Mellitus (RAMP-DM) at 12 months follow-up in terms of biomedical outcomes, observed cardiovascular events and predicted long-term cardiovascular risks in the primary care setting. This was the first study to provide imperative translational evidence of risk-stratification and risk-specific management for diabetes in the real world primary care setting.

## Methods

### Study design

We conducted a longitudinal comparative study to compare the changes of biomedical measurements, observed cardiovascular events and cardiovascular risks at 12 months follow-up using a difference-in-differences approach between the RAMP-DM group and the usual care group.

### Setting of RAMP-DM

The RAMP-DM is a territory-wide program which intends to cover all the people with diabetes under the care of public General Out-Patient Clinics (GOPCs) in Hong Kong. GOPCs provide primary care for about 190,000 people with diabetes, which account for about 60% of all the people with diabetes in Hong Kong. From August 2009 to June 2010, the RAMP-DM was piloted in four of the seven clusters in Hong Kong. Details of the RAMP-DM program have been reported elsewhere [17]. In brief, all people with diabetes under GOPCs care are eligible to be enrolled in the RAMP-DM. The enrolled subjects underwent a series of assessment of risk factors and potential existing diabetic complications when they entered the program, which included measurements of basic parameters (body mass index, waist circumference, blood pressure, etc.), tests of biomedical parameters (HbA_1_c, full lipid profile, renal function, etc.), and examinations of eyes and feet. Based on the results of these screenings and history of previous complications, RAMP-DM participants were stratified into ‘very high’, ‘high’, ‘medium’ and ‘low’ risk groups according to the JADE classification [14]. For RAMP-DM participants with different risk stratifications, different management strategies were provided by a team of multidisciplinary healthcare professionals, which includes associate consultants in family medicine, GOPCs doctors, advanced practice nurses, registered nurses, and allied health professionals (namely optometrist, dietitian, podiatrist and physiotherapist). Enrolled patients were also eligible to be invited to join the Patient Empowerment Program (PEP), which is a structured education program delivered by two non-governmental organizations to enhance patients’ knowledge and skill on diabetes self-care management. In summary, the interventions for RAMP-DM participants included nurse assessment (including risk stratification), and would be arranged in addition to care from GOPCs doctors, to have nurse intervention, associate consultant intervention, allied-health professional intervention and PEP according to risk levels and need.

Patients under usual care, on the other hand, will be continuously managed solely by GOPCs doctors without risk assessment and stratification. These patients could still be referred to allied health professionals and PEP at their doctors’ discretion.

### Subjects

To detect a 5% between group difference in the proportion of achieving the clinical treatment target (HbA_1_c <7%), with 80% power and 95% confidence interval, 1,248 subjects were needed in each group. A sample of 1,248 RAMP-DM participants (312 from each of the 4 clusters) was randomly selected out of 18,492 subjects, who were enrolled in RAMP-DM between August 2009 and June 2010. A group of 1,248 people with diabetes who attended GOPC but were not enrolled in RAMP-DM during this period were matched by age, sex and HbA1c level as a control group. The baseline dates for RAMP-DM participants are the dates when they received nurse assessment. For usual care group, the baseline date is set as 30 June 2010. The follow-up period for both groups is 12 months since their respective baseline dates.

The ethics approval for this study was granted by the Institutional Review Board of The University of Hong Kong and relevant clusters.

### Outcome measures

The primary outcomes of this study were the changes in HbA_1_c, observed cardiovascular events and predicted 10-year cardiovascular risks over 12 months follow-up. The secondary outcomes were changes in blood pressure, lipid profiles and BMI. We assessed the changes in biomedical outcomes by mean values as well as the proportions of subjects reaching treatment targets in HbA_1_c (<7%), blood pressure (<130/80 mmHg) and LDL-C (<2.6 mmol/L) recommended by Hong Kong local framework [18]. The 10-year predicted cardiovascular risks were estimated by the Framingham risk function for total cardiovascular disease (CVD) risk [19], and the UKPDS risk engines for coronary heart disease (CHD) [20] and stroke [21], respectively. Demographic data, biomedical outcomes, disease history, drug treatment and RAMP-DM intervention data were extracted from the population-based health and intervention linked database (Clinical Management System) owned by Hospital Authority. To avoid the impact of aging on CVD risks, we applied the age at baseline to calculate the CVD risks at both baseline and 12 months.

### Data analysis

We used the mean and standard deviation to describe continuous variables and the categorical variables were summarized by counts and percentages. Paired *t*-test was employed to compare the paired differences in biomedical measurements and cardiovascular risks between baseline and 12 months for all subjects. The differences in proportions of subjects achieving treatment targets between baseline and 12 months were examined by the McNemar test. We applied the Chi-square test to investigate the between group differences in the incidences of cardiovascular events.

The difference-in-differences approach was used to test the between group differences in the changes of biomedical measurements, cardiovascular risks and target achievement rates. In other words, we calculated the within-group changes over 12 months follow-up for outcomes of interest, and compared the within-group changes between RAMP-DM and control groups by *t*-test in unadjusted analysis.

To adjust for potential confounders, we constructed three multiple linear regression models to adjust the baseline parameters and drug treatment. Model 1 adjusted the baseline parameters only, model 2 adjusted the baseline parameters and drug treatment at baseline, and model 3 adjusted the baseline parameters, drugs treatment at both baseline and 12 months follow-up.

To further investigate the effects of each component of the RAMP-DM interventions on the changes of biomedical outcomes and cardiovascular risks, we applied multiple linear regressions adjusting for the baseline parameters, and drug treatment among RAMP-DM participants.

We used STATA Version 12.0 (StataCorp LP. College Station, Texas, U.S.) to conduct all the data analyses, and a *P*-value less than 0.05 was considered as statistically significant.

## Results

We excluded 80 control subjects with existing CVD at baseline and 96 control subjects who were enrolled in RAMP-DM over 12 months follow-up. The same number of case–control pairs from RAMP group were excluded as well (including 54 subjects with previous CVD). The baseline characteristics of RAMP-DM group and the usual care group are shown in Table 1.

**Table 1:** **Baseline characteristics of participants in RAMP-DM group and usual care group**

	**RAMP-DM (N = 1072)**	**Usual care (N = 1072)**	
	**Mean ± SD or n(%)**		***P*** **-value** ^**a**^
*Demographic*			
Age (year)	64.3 ± 10.9	65.3 ± 11.7	0.056
Male	534 (49.8%)	534 (49.8%)	1.000
Smokers	106 (9.9%)	90 (8.4%)	0.231
Cluster information			0.767
HKEC	268 (25.0%)	278 (25.9%)	
KCC	270 (25.2%)	274 (25.6%)	
KWC	256 (23.9%)	262 (24.4%)	
NTEC	278 (25.9%)	258 (24.1%)	
*Clinical*			
T2DM	1064 (99.5%)	1006 (93.8%)	<0.001
Duration of DM (year)	7.9 ± 6.2	7.9 ± 6.6	0.759
HbA_1_c (%)	7.26 ± 1.17	7.13 ± 1.17	0.033
TC(mmol/L)	5.05 ± 0.96	4.87 ± 0.94	<0.001
HDL-C (mmol/L)	1.23 ± 0.34	1.23 ± 0.33	0.830
LDL-C (mmol/L)	3.10 ± 0.80	2.89 ± 0.79	<0.001
Triglycerides (mmol/L)	1.63 ± 1.08	1.56 ± 1.00	0.2495
SBP ≤ 140 mmHg	615 (58.7%)	634 (69.3%)	<0.001
DBP ≤ 80 mmHg	700 (66.8%)	687 (75.1%)	<0.001
SBP/DBP ≤ 140/80 mmHg	538 (58.8%)	491 (46.9%)	<0.001
*No(%) reaching treatment target*			
HbA_1_c < 7%	467 (50.54%)	384 (54.9%)	0.079
SBP < 130 mmHg	365 (34.8%)	412 (45.0%)	<0.001
DBP < 80 mmHg	700 (66.8%)	687 (75.1%)	<0.001
SBP/DBP < 130/80 mmHg	323 (30.8%)	381 (41.6%)	<0.001
LDL-C < 2.6 mmol/L	154 (27.3%)	132 (39.4%)	<0.001
*Predicted cardiovascular risk (%)*			
Framingham 10-year CVD risk^b^	35.18 ± 20.68	33.32 ± 20.16	0.0882
UKPDS 10-year CHD risk^c^	23.25 ± 16.29	22.00 ± 15.90	0.177
UKPDS 10-year stroke risk^d^	14.51 ± 15.21	15.06 ± 15.79	0.6439

^a^Independent *t*-test for continuous variables and Chi square test for dichotomous variables.

^b^Predicted by the Framingham cardiovascular risk function.

^c^Predicted by the UKPDS CHD risk engine.

^d^Predicted by the UKPDS stroke risk engine.

BMI, body mass index; CVD, cardiovascular disease; CHD, coronary heart disease; SBP, systolic blood pressure; DBP, diastolic blood pressure; DM, diabetes mellitus; HbA_1c_, glycated hemoglobin; HDL-C, high density lipid cholesterol; HKE, Hong Kong East Cluster; KCC, Kowloon Central Cluster; KWC, Kowloon West Cluster; LDL-C, low density lipid cholesterol; NTEC, New Territories East Cluster; TC, total cholesterol. T2DM, Type 2 diabetes mellitus.

At baseline (Table 1), the proportions of patients reaching treatment target (HbA_1_c < 7%) was similar between RAMP-DM group and usual care group (*P* = 0.079). RAMP-DM participants had significantly higher levels in TC (*P* < 0.001), LDL-C (*P* < 0.001) and blood pressure (*P* < 0.001), resulting in smaller proportions of patients reaching treatment targets in LDL-C (*P* < 0.001) and blood pressure (*P* < 0.001). The predicted 10-year total CVD risk by the Framingham risk function, 10-year risks of CHD and stroke by the UKPDS risk engines were similar (*P* = 0.088, *P* = 0.177 and *P* = 0.644, respectively).

### Drug treatment and RAMP-DM interventions

Table 2 summarizes the drug treatment and RAMP-DM interventions for included subjects. At baseline, a significantly higher proportion in the usual care group were using insulin (*P* < 0.001), and this trend continued at 12 months. Among RAMP-DM participants, on average 39.2% and 13.3% subjects received nurse intervention and associate consultant intervention respectively. Patients under usual care were also eligible for PEP and allied health professionals consultation, but the proportions having these interventions were quite small (2.6% and 1.1%, respectively).

**Table 2:** **Treatment modalities of participants in RAMP-DM group and usual care group at baseline and 12 months**

	**RAMP-DM (N = 1072)**	**Usual care (N = 1072)**	
	**n(%)**		**P-value** ^**a**^
Treatment Modalities at baseline			
On oral glucose-lowering drugs	949 (88.5%)	941 (87.8%)	0.593
On insulin	17 (1.59%)	76 (7.1%)	<0.001
On anti-hypertensive drugs	835 (77.9%)	818 (76.3%)	0.382
On lipid-lowering drugs	866 (80.8%)	880 (82.1%)	0.437
Treatment Modalities at 12 months			
On oral glucose-lowering drugs	967 (90.2%)	949 (88.5%)	0.207
On insulin	63 (5.9%)	115 (10.7%)	<0.001
On anti-hypertensive drugs	871 (81.3%)	852 (79.5%)	0.302
On lipid-lowering drugs	935 (87.2%)	949 (88.5%)	0.354
Enrolled in PEP	77 (7.2%)	28 (2.6%)	<0.001
Receiving nurse assessment	1072 (100%)	--	--
Receiving nurse intervention	766 420 (39.2%)	--	--
Receiving associate consultant intervention	143 (13.3%)	--	--
Receiving allied health professionals intervention	190 (17.8%)	12 (1.1%)	<0.001

^a^Chi square test.

PEP, patient empowerment program.

### Observed cardiovascular events

Table 3 shows the incidences of observed cardiovascular events over 12 months follow-up. A total of 12 (1.21%) and 31 (2.89%) CVD events were observed in RAMP-DM group and control group respectively. Compared to the control subjects, RAMP-DM participants had significantly lower incidences in CHD (−1.49%, *P* < 0.001) and total CVD (−1.77%, *P* = 0.003).

**Table 3:** **Observed one-year cardiovascular events over 12-month follow-up**

	**RAMP-DM (N = 1072)**	**Usual care (N = 1072)**	**Between groups differences**	
**Event**	**n(%)**	**n(%)**	**Estimate**	**P-value** ^**a**^
CHD	2 (0.19%)	18 (1.68%)	−1.49%	<0.001
Stroke	10 (0.93%)	13 (1.21%)	−0.28%	0.529
Total CVD (CHD + stroke)	12 (1.21%)	31 (2.89%)	−1.77%	0.003

^a^Chi Square test.

CVD, cardiovascular disease; CHD, coronary heart disease.

### Biomedical outcomes

Over 12 months follow-up, RAMP-DM subjects showed significant improvements in the control of HbA_1_c (−0.11%, *P* < 0.01), lipid profiles (TC, −0.27 mmol/L, *P* < 0.01; LDL-C, −0.31 mmol/L, *P* < 0.01, HDL-C,0.04 mmol/L, *P* < 0.01; Triglyceride, −0.09, *P* < 0.05), blood pressure (SBP,-4.20 mmHg, *P* < 0.01; DBP,-2.53 mmHg, *P* < 0.01) and BMI (−0.32 kg/m^2^, *P* < 0.01) We observed increase in the proportions of reaching the treatment targets in HbA_1_c (4.11%, *P* < 0.05), LDL-C (17.52%, *P* < 0.01) and blood pressure (5.34%, *P* < 0.01) (Table 4). In the control group, a significant increase in HbA_1_c was observed compared to baseline (0.10%, *P* < 0.05). Compared to the control subjects, RAMP-DM participants showed significantly larger improvements in HbA_1_c (−0.20%, *P* < 0.01), HDL-C (0.02 mmol/L, *P* < 0.05) and blood pressure (SBP,-3.62 mmHg, *P* < 0.01; DBP,-1.73 mmHg, *P* < 0.01). After adjusting for baseline parameters and drug treatment, the differences in the changes of HbA_1_c and DBP were still significant (Table 4).

**Table 4:** **Comparison of changes in biomedical outcomes and cardiovascular risks between RAMP-DM group and usual care group at 12 months**

	**Paired difference (12 months – baseline)**		**Unadjusted D-in-D**	**Model 1**	**Model 2**	**Model 3**
**Variables**	**RAMP-DM (N = 1072) Mean (95% CI)**	**Usual care (N = 1072) Mean (95% CI)**	**Estimate (95% CI)**	**Coefficients (95% CI)**	**Coefficients (95% CI)**	**Coefficients (95% CI)**
HbA_1_c (%)	−0.11** (−0.18, −0.04)	0.10* (0.01, 0.17)	−0.20** (−0.30,-0.09)	−0.14** (−0.23,-0.05)	−0.11* (−0.21, −0.02)	−0.11* (−0.20,-0.02)
TC (mmol/L)	−0.27** (−0.33, −0.21)	−0.25** (−0.32, −0.19)	−0.02 (−0.10,0.07)	−0.07 (−0.15,0.01)	−0.07 (−0.15,0.01)	−0.06 (−0.12,0.03)
LDL-C (mmol/L)	−0.31** (−0.38, −0.24)	−0.21** (−0.29, −0.13)	−0.10 (−0.21, 0.01)	−0.03 (−0.07, 0.12)	−0.02 (−0.12, 0.07)	−0.02 (−0.08, 0.11)
HDL-C (mmol/L)	0.04** (0.03, 0.05)	0.02* (0.002, 0.03)	0.02* (0.01, 0.04)	0.02 (−0.005, 0.04)	0.02 (−0.004, 0.04)	0.02 (−0.004, 0.04)
Triglyceride (mmol/L)	−0.09* (−0.18, −0.01)	−0.10** (−0.17, −0.03)	0.02 (−0.14, 0.11)	0.04 (−0.07, 0.16)	0.04 (−0.08, 0.16)	0.04 (−0.08, 0.16)
SBP (mmHg)	−4.20** (−5.25, −3.04)	−0.58 (−0.65, 1.81)	−3.62** (−5.31, −1.93)	−0.21 (−1.58, 1.16)	−0.32 (−1.70, 1.04)	−0.40 (−1.73, 0.93)
DBP (mmHg)	−2.53** (−3.18, −1.88)	−0.60 (−0.06, 1.26)	−1.73** (−2.10, −1.16)	−0.89* (−1.66, −0.11)	−0.95* (−1.73, −0.18)	−0.94* (−1.72, −0.16)
BMI (kg/m^2^)	−0.32** (−0.41, −0.23)	−0.21** (−0.33, −0.07)	−0.11 (−0.26, 0.04)	−0.07 (−0.22, 0.08)	−0.06 (−0.21, 0.09)	−0.06 (−0.21, 0.09)
Percentage reaching treatment target (%)						
HbA_1_c < 7%	4.11* (0.63, 7.60)	−1.29 (−5.32, 2.75)	5.40* (0.25, 10.55)	4.24 (−0.79, 9.27)	3.29 (−1.79, 8.37)	3.16 (−1.93, 8.24)
SBP < 130 mmHg	5.34** (1.67, 9.02)	2.19 (−1.79, 6.16)	3.16 (−2.25, 8.57)	4.08 (−1.00, 9.16)	3.74 (−1.35, 8.83)	3.71 (−1.39, 8.81)
DBP < 80 mmHg	8.87** (5.61, 12.14)	0.98 (−2.24, 4.20)	7.89** (3.42, 12.36)	4.49* (0.34, 8.63)	4.58* (0.40, 8.75)	4.57* (0.39, 8.74)
SBP/DBP < 130/80 mmHg	6.20** (2.63, 9.77)	0.43 (−3.43, 4.31)	5.77* (0.51, 11.02)	0.97 (−3.98, 5.93)	7.25 (−4.25, 5.70)	0.74 (−4.23, 5.73)
LDL-C < 2.6 mmol/L	17.52** (12.80, 22.24)	11.94** (5.53, 18.35)	5.58 (−1.99, 13.15)	0.07 (−7.47, 7.62)	0.17 (−7.40, 7.74)	0.50 (−7.00, 8.00)
Predicted cardiovascular risk (%)						
Framingham 10-year CVD risk^a^	−3.93** (−4.63, −3.32)	−1.87** (−2.70, −1.05)	−2.06** (−3.14, −0.98)	−1.70** (−2.69, −0.68)	−1.67 (−2.52, 0.73)	−1.73 (−2.65, 0.75)
UKPDS 10-year CHD risk^b^	−3.00** (−3.54, −2.44)	−1.55** (−2.19, −0.92)	−1.43** (−2.23, −0.58)	−1.20** (−2.00, −0.43)	−1.22* (−2.02, −0.45)	−1.25* (−2.05, −0.45)
UKPDS 10-year stroke risk^c^	−1.17** (−1.49, −0.85)	−0.46 (−0.01, 0.01)	−0.71* (−1.28, −0.15)	−0.96** (−1.50, −0.49)	−1.00** (−1.51, −0.49)	−0.77** (−1.30, −0.23)

*P-value < 0.05; **P-value <0.01.

^a^Predicted by the Framingham cardiovascular risk function.

^b^Predicted by the UKPDS CHD risk engine.

^c^Predicted by the UKPDS stroke risk engine.

D-in-D, difference-in-differences; BMI, body mass index; CVD, cardiovascular disease; CHD, coronary heart disease; SBP, systolic blood pressure; DBP, diastolic blood pressure; DM, diabetes mellitus; HbA_1c_, glycated hemoglobin; HDL-C, high density lipid cholesterol; LDL-C, low density lipid cholesterol; TC, total cholesterol.

Model 1, adjusted for age, sex, smoking status, duration of DM, type of DM, and baseline value of the outcome measure; Model 2, adjusted for age, sex, smoking status, duration of DM, type of DM, baseline value of the outcome measure, and four types of drugs at baseline; Model 3, adjusted for age, sex, smoking status, duration of DM, type of DM, baseline value of the outcome measure, four types of drugs at baseline and drugs at 12 months.

### Predicted cardiovascular risks

After 12 months of follow-up, significant decreases in predicted total CVD risks and CHD risks were observed in both RAMP-DM and control groups (Table 4). The control group failed to achieve improvement in stroke risk. The unadjusted difference-in-differences of cardiovascular risks were significant in total CVD risk (−2.06%, *P* < 0.01), as well as the specific CHD (−1.43%, *P* < 0.01) and stroke risk (−0.71%, *P* < 0.05), and these differences remained significant after adjusting for baseline parameters. After adjusting for baseline parameters and drug treatments, the RAMP-DM participants still showed larger reductions in the risks of CHD and stroke predicted by the UKPDS risk engines.

### Impact of individual RAMP-DM intervention on biomedical outcomes and predicted CVD risks

After observing improvements in biomedical outcomes and reductions in CVD risks in RAMP-DM participants, multiple linear regression analyses were conducted to further investigate the impact of each component of RAMP-DM interventions on the improvements in clinical outcomes among RAMP-participants. The results are shown in Table 5. After adjusting for baseline parameters, drug treatment at baseline and 12 months, associate consultant intervention was associated with decreases in LDL-C (*P* < 0.05), triglyceride (*P* < 0.05) and CHD risk (*P* < 0.01). PEP was associated with a decrease in BMI, but a paradoxical increase in DBP.

**Table 5:** **The impact of each component of RAMP-DM intervention on the change in biomedical measurements and cardiovascular risks**

	**Multiple linear regression** ^**d**^			
	**Advanced Nurse Intervention**	**Associate consultant intervention**	**Allied health professionals consultation**	**PEP**
	**Coefficients (95% CI)**			
Change in HbA_1_c (%)	−0.01 (−0.12, 0.12)	−0.12(−0.34, 0.107)	−0.19(−0.40, 0.01)	−0.19(−0.42, 0.05)
Change in TC	0.05 (−0.06, 0.16)	−0.33 (−0.50, −0.16)**	−0.09 (−0.27, 0.09)	−0.32 (−0.51, −0.13)**
Change in HDL-C	0.01 (−0.02, 0.04)	0.01 (−0.04, 0.04)	−0.03 (−0.08, 0.02)	−0.03 (−0.07, 0.02)
Change in LDL-C	0.09 (−0.04, 0.22)	−0.26 (−0.46, −0.06)*	−0.11 (−0.19, 0.17)	−0.22 (−0.45, 0.02)
Change in Triglyceride	0.08 (−0.12, 0.27)	−0.40 (−0.71, −0.09)*	−0.12 (−0.45, 0.20)	−0.26 (−0.61, 0.08)
Change in SBP	−0.88 (−2.79, 1.03)	2.18 (−0.70, 5.06)	−2.45 (−5.70, 0.81)	−1.11 (−4.51, 2.29)
Change in DBP	−0.35 (−1.48, 0.77)	0.56 (−1.15, 2.26)	−0.72 (−2.62, 1.17)	2.43 (0.42, 4.43)*
Change in BMI	−0.01 (−0.20, 0.19)	−0.07 (−0.39, 0.22)	−0.18 (−0.47, 0.10)	−0.28 (−0.60, −0.03)
Change in 10-year CVD risk^a^	−0.15 (−1.10, 1.41)	−1.35 (−3.28, 0.01)	−1.59 (−3.64, 0.45)	−1.29 (−3.47, 0.01)
Change in 10-year CHD risk^b^	−0.46 (−1.54, 0.62)	−2.77 (−4.54, 1.00)**	−1.31 (−3.02, 0.04)	−1.72 (−3.81, 0.38)
Change in 10-year stroke risk^c^	−0.04 (−1.02, 0.26)	0.99 (−0.20, 0.01)	−0.05 (−1.68, 0.01)	−0.02 (−1.30, 0.96)

*P < 0.05; **P < 0.01.

^a^Predicted by the Framingham cardiovascular risk function.

^b^Predicted by the UKPDS CHD risk engine.

^c^Predicted by the UKPDS stroke risk engine.

^d^Adjusted for confounding variables including age, sex, smoking status, duration of DM, type of DM, baseline value of the outcome measure, drugs at baseline and drugs at 12 months.

BMI, body mass index; CVD, cardiovascular disease; CHD, coronary heart disease; SBP, systolic blood pressure; DBP, diastolic blood pressure; DM, diabetes mellitus; HbA_1c_, glycated hemoglobin; HDL-C, high density lipid cholesterol; LDL-C, low density lipid cholesterol; TC, total cholesterol; PEP, patient empowerment program.

## Discussion

To the best of our knowledge, this is the first study to evaluate the effectiveness of a risk-stratification management approach involving multidisciplinary interventions for people with diabetes in the real-world primary care setting. This study found that the RAMP-DM intervention led to lower incidence of cardiovascular events and significant improvements in HbA_1_c and predicted cardiovascular risks compared to the usual care. Further investigation of the effects of each RAMP-DM intervention component found that, after adjusting for potential confounders, associate consultant intervention was associated with improvements in lipid control and predicted CHD risk, while PEP was related to decreases in BMI.

Compared to the previous study on risk stratification and intervention in staff-model primary care clinics by Clark et al. [12], the magnitude of improvement in HbA_1_c in our study was smaller. In our study, there was a 0.2% net decrease of HbA_1_c in RAMP-DM group, while Clark’s study showed about 0.35% between group differences in the changes of HbA1c after 12-month follow-up. Our subjects were much less severe at baseline with an average HbA1c of 7.2%, whereas the mean HbA_1_c in Clark’s study was above 8.5%. The RAMP-DM is designed to cover all people with diabetes in the primary care in Hong Kong; therefore, we randomly selected subjects to assess the effectiveness of RAMP-DM in general instead of selecting severer cases deliberately. We observed 5.40% more subjects under RAMP-DM reaching treatment target (HbA1c < 7.0%), which addressed the clinical benefits of RAMP-DM.

RAMP group had a greater increase in the proportions of reaching HbA1c < 7%, and SBP/DBP < 130/80 mmHg compared to usual care group, but after adjusting for the baseline parameters and drug treatment, the differences became insignificant, although the results were still favoring RAMP-DM participants. Male sex and no history of myocardial infarction were found to be associated with uncontrolled blood pressure [22]. As all of our study subjects were without cardiovascular complications at baseline and gender was well matched between groups, it was likely that the baseline parameters and drug treatment affected the outcomes. Drug treatment and duration of disease were indicators of diabetes severity. Moe et.al found that compared to people with diabetes and without medication, these on medication subjects had higher risk of cardiovascular death [23]. For severer subjects at baseline, doctors might provide them with more intensive care, no matter they were enrolled in RAMP-DM or not, leading to bigger improvement. Also, regression to the mean might lead to bigger reduction for those with higher baseline HbA_1_c and SBP levels. In addition, subjects in control group were also eligible to be referred to some of the services in the RAMP-DM intervention package (allied health professionals and PEP) if necessary, which might bias the effects of RAMP-DM towards null.

The RAMP-DM group observed fewer coronary heart disease and total cardiovascular events compared to the control group during 12 months follow-up. This is consistent with the findings of the improvement of HbA_1_c and predicted cardiovascular risks in RAMP-DM group. A recent study shows that the increase in HbA_1_c level is significantly associated with the incidence of coronary heart disease during 6 years follow-up [24]. To validate the association in our study, longer follow-up period is needed.

We employed the Framingham cardiovascular risk function developed for primary care [19] to assess the longer term effects in total CVD risk, and applied the UKPDS risk engines [20,21] to predict the changes in CHD and stroke risks. Although we found that RAMP-DM participants showed significantly greater improvement in the total CVD risk, the differences were not significant after adjusting for drug treatment, while the differences in CHD and stroke risk predicted by the UKPDS risk engines remained significant. Our previous study found that the UKPDS risk engine is more sensitive to detect differences in CHD risk in Chinese people with diabetes [25], as it was developed for people with diabetes specifically. Moreover, the previous study showed that the CHD risk predicted by the UKPDS risk function showed excellent convergent validity with the JADE risk function that was developed in Chinese people with diabetes [25,26]. We could not use the JADE risk function for the estimation of CVD risk in this study because many required parameters such as estimated glomerular filtration rate and urine albumin:creatinine ratio were missing in many subjects.

Very few studies on DM management measured cardiovascular risk reduction as an outcome. Most studies only reported changes in blood pressure and lipid profiles in addition to HbA_1_c. Comprehensive cardiovascular risk management is getting increasing attention in diabetes care [5,27]. The RAMP-DM provided personalized risk-stratification based care to people with diabetes by multidisciplinary health care professionals, which promoted the concept of cardiovascular risk management and facilitated the optimization of medical resources.

The RAMP-DM addressed four interralated components of the Chronic Care Model [10], with multidisciplinary management involving doctors, nurses and allied health professionals. By exploration on individual intervention components among RAMP-DM participants, we found that nurse intervention alone was not associated with any improvements in biomedical outcomes and cardiovascular risks.

Previous trials on nurses led interventions resulted in inconsistent findings in changes of HbA_1_c. The PEACH study in the Australia primary care setting delivering telephone coaching on medication goals by practice nurses failed to achieve improvement in HbA_1_c and other relevant biomedical measures [28]. A Spain based standardized language in nursing care plans [29] and a U.S. based nurse care management also showed no improvements in HbA_1_c [30]. However, a nurse-led telephone coaching intervention in the U.S. found significant reductions in HbA_1_c [31]. Lacking of prescribing rights is likely to limit the role of nurses in diabetes management, thus affect the benefits of sole nurses intervention [28].

On the other hand, most multidisciplinary interventions involving at least nurses and physicians found favoring results on blood glucose control. A multidisciplinary intervention for patients with HbA_1_c higher than 10% in Israel found the intervention group had significant decrease in HbA_1_c after six months. This multidisciplinary team contained diabetologist, the dietician and the diabetes nurse educator [32]. Positive results were also found in similar multidisciplinary interventions in Taiwan [33], the U.S. [34] and France [35]. Most of these multidisciplinary interventions included diabetic education sessions. A symposium convened by American Association of Diabetes Educators acknowledged that the most effective education programs occurred within multidisciplinary teams [36].

The PEP was associated with improvement in BMI, and had favoring effects on HbA_1_c, lipid profiles and SBP. The small number of subjects enrolled in PEP (7.2%) might be insufficient to detect significant changes. An independent study on the effectiveness of PEP confirmed these favoring results [37]. A RCT was designed to compare the effects of long-term (2 years) education program with initial education only in France. The results are yet to report [38].

There are several limitations of this study. First, since it is not a RCT, some unknown potential confounders might affect the results. However, a study found that the positive effects of interventions in controlled trial settings could not be replicated in real-world primary care settings [39]. Second, the lipid profiles and blood pressure between the RAMP-DM and usual care groups were not well matched at baseline, which might affect the changes over 12 months. Third, the lack of blinding of clinicians and patients is the inherent limitation of population based clinical interventions. Fourth, the Framingham risk function and the UKPDS risk engines were developed in western population, and the UKPDS risk engines were developed in subjects with type 2 diabetes. We had less than 1% subjects with type 1 diabetes, which might affect the accuracy of the predicted CHD and stroke risks. As cardiovascular events need time to develop, the follow-up period of one year was not long enough for us to validate the predicted CVD risk with observed cardiovascular events.

## Conclusions

This longitudinal comparative study in a pragmatic primary care setting found that a multidisciplinary risk assessment and management program for people with diabetes (RAMP-DM) significantly reduced HbA_1_c, observed CVD events and predicted 10-year cardiovascular risks over 12 months follow-up. The encouraging results support the risk stratification and multidisciplinary approach for the management of diabetic patients. A further study focusing on the longer term effects of RAMP-DM in terms of the cardiovascular risk control and the effects of the frequency of interventions will be conducted at three years follow-up.

## Abbreviations

CHD: Coronary heart disease
CVD: Cardiovascular Disease
DM: Diabetes mellitus
GOPC: General Out-Patient Clinics
GP: General practitioner
JADE: Joint Asia Diabetes Evaluation Program
PEP: Patient Empowerment Program
RAMP-DM: Risk Assessment and Management Program for Patients with Diabetes Mellitus
